# Overcoming barriers to engaging socio-economically disadvantaged populations in CHD primary prevention: a qualitative study

**DOI:** 10.1186/1471-2458-10-391

**Published:** 2010-07-02

**Authors:** Christopher Harkins, Rebecca Shaw, Michelle Gillies, Heather Sloan, Kate MacIntyre, Anne Scoular, Caroline Morrison, Fiona MacKay, Heather Cunningham, Paul Docherty, Paul MacIntyre, Iain N Findlay

**Affiliations:** 1 Public Health and Health Policy, University of Glasgow, 1 Lilybank Gardens, Glasgow, G12 8RZ, UK; 2 Greater Glasgow & Clyde NHS Board, Dalian House, 350 St Vincent Street, Glasgow, G3 8YZ, UK; 3 Renfrewshire Community Health Partnership, NHS Greater Glasgow & Clyde, Renfrewshire House, Paisley, PA1 1UJ, UK; 4 NHS Greater Glasgow & Clyde Health Information & Technology, Development Department, Westward House, St James Street, Paisley, PA3 2HL, UK; 5 Department of Cardiology, Royal Alexandra Hospital, Paisley, PA2 9PN, UK

## Abstract

**Background:**

Preventative medicine has become increasingly important in efforts to reduce the burden of chronic disease in industrialised countries. However, interventions that fail to recruit socio-economically representative samples may widen existing health inequalities. This paper explores the barriers and facilitators to engaging a socio-economically disadvantaged (SED) population in primary prevention for coronary heart disease (CHD).

**Methods:**

The primary prevention element of Have a Heart Paisley (HaHP) offered risk screening to all eligible individuals. The programme employed two approaches to engaging with the community: a) a social marketing campaign and b) a community development project adopting primarily face-to-face canvassing. Individuals living in areas of SED were under-recruited via the social marketing approach, but successfully recruited via face-to-face canvassing. This paper reports on focus group discussions with participants, exploring their perceptions about and experiences of both approaches.

**Results:**

Various reasons were identified for low uptake of risk screening amongst individuals living in areas of high SED in response to the social marketing campaign and a number of ways in which the face-to-face canvassing approach overcame these barriers were identified. These have been categorised into four main themes: (1) processes of engagement; (2) issues of understanding; (3) design of the screening service and (4) the priority accorded to screening. The most immediate barriers to recruitment were the invitation letter, which often failed to reach its target, and the general distrust of postal correspondence. In contrast, participants were positive about the face-to-face canvassing approach. Participants expressed a lack of knowledge and understanding about CHD and their risk of developing it and felt there was a lack of clarity in the information provided in the mailing in terms of the process and value of screening. In contrast, direct face-to-face contact meant that outreach workers could explain what to expect. Participants felt that the procedure for uptake of screening was demanding and inflexible, but that the drop-in sessions employed by the community development project had a major impact on recruitment and retention.

**Conclusion:**

Socio-economically disadvantaged individuals can be hard-to-reach; engagement requires strategies tailored to the needs of the target population rather than a population-wide approach.

## Background

Despite sustained reductions in coronary heart disease (CHD) incidence and mortality over the last decade, CHD remains the commonest cause of premature death in men and women in Scotland [1]. Research has suggested that a significant proportion of incident CHD is attributable to modifiable risk factors such as smoking, diet and level of physical activity [2]. This recognition that CHD is a largely preventable disease has focused health policy, both in the UK and elsewhere, on primary prevention [3,4].

Research has also shown that socioeconomic deprivation (SED) is associated with excess CHD morbidity and mortality [5]. For the most deprived populations in Scotland, premature mortality from CHD actually increased over the period 2003-2006 in contrast to other groups [1]. This excess risk is partially mediated through the distribution of known CHD risk factors in deprived populations [5] and it has been estimated that equal uptake of effective primary prevention across all socioeconomic groups in the UK would eliminate almost 70% of the excess CHD mortality experienced by socio-economically disadvantaged individuals [6]. However, prevention interventions that fail to engage with deprived populations may actually serve to widen health inequalities. This presents policy makers and health care practitioners with the challenge of trying to implement effective primary prevention interventions that engage and recruit across all socio-economic strata.

The elements of health promotion strategies that are most effective for recruiting and engaging participants are not well understood [7,8] and, in particular, there is a paucity of good quality research examining the most effective strategies to engage 'hard-to-reach', deprived populations in preventative medicine. Yancey et al [9] conducted a general assessment of the available literature and identified a number of issues that make studies challenging to compare and findings difficult to generalise. These include differences in the reporting of recruitment, enrolment, and retention information; inconsistencies in the use of terminology across studies and the complexity of the literature which covers disparate samples of socio-demographic compositions, different diseases and study types. Literature specifically concerning recruitment into CHD primary prevention is especially scarce. Fitzgibbon et al [10] report the labour-intensive nature of effective recruitment within underserved communities in two separate CHD primary prevention programmes. Neighbourhood canvassing, presentations and telephone recruitment methods were cited as successful approaches; however the authors stress the importance of tailoring recruitment efforts to the needs, experiences and environment of the target population. In another CHD primary prevention programme, King et al [11] adopted two recruitment efforts; a random-digit-dial telephone survey and a community media campaign. This study reports few differences in the demographics of the recruitment yield from each approach; however the telephone survey recruitment was particularly successful in recruiting smokers and persons with other cardiovascular risk factors. Furthermore, counter to expectations, subsequent programme adherence rates did not differ by recruitment source.

The primary prevention element of the Have a Heart Paisley (HaHP) study offered risk screening to individuals aged 45-60 years old, without a prior history of heart disease and registered with a general practitioner (GP) in Paisley, in the West of Scotland [12]. The programme employed two approaches to engage with the community (a) a widespread social marketing campaign and (b) a community development project adopting primarily face-to-face canvassing. In this paper we present the results of a qualitative study that employed focus groups to explore the perceived barriers and facilitators experienced by individuals from a socio-economically disadvantaged population to engaging with a CHD primary prevention intervention.

## Methods

### Setting

HaHP was a Scottish National Demonstration Project undertaken in Paisley (population approximately 85,000) in the West of Scotland [12]. Between 2005 and 2008, as part of the project's primary prevention intervention, CHD risk screening was offered to all individuals (aged 45-60 years old) who were registered with a general practitioner in Paisley and free from CHD at the time of study enrolment. Following screening, individuals found to be at increased risk for CHD were invited to take part in a health coaching programme to support lifestyle and behavioural change.

Two distinct approaches to recruitment were adopted within the project. The first approach utilised a social marketing campaign [13] via the local media and community-based events to promote awareness of the intervention alongside mass mailings to invite eligible individuals to participate. This approach was adopted across the entire Paisley catchment area. A second technique, employing a community development approach [14], was undertaken specifically within a local area with extreme socio-economic deprivation (Ferguslie Park). This second approach involved more direct attempts to recruit participants, including face-to-face canvassing within prominent community venues, door-to-door cold calling and peer referral.

### Participants

11,273 individuals were identified as eligible (aged 45-60, enrolled with a GP in Paisley and without a history of CHD) from approximately 85,000 GP records using the HaHP Chronic Disease Register (CDR) [15] and invited to attend for CHD risk screening as part of the social marketing campaign.

Table 1 shows the socio-demographic breakdown of the target populations in terms of the Scottish Index of Multiple Deprivation (SIMD), which uses data about current income, employment, health, education skills and training, geographic access to services, housing and crime to provide a measure of local area deprivation. Table 1 also includes the proportion of individuals who accepted screening; detailed for both recruitment approaches. Furthermore the social marketing target population and recruitment yield for 'All Paisley' and 'Ferguslie Park' geographical areas are detailed in table 1.

**Table 1:** Socio-demographic profile of HaHP target population and individuals accepting screening by recruitment method and study geographical areas

	Social Marketing Recruitment (All Paisley and Ferguslie Park area of Paisley)						Community Development Recruitment (Ferguslie Park area of Paisley)		
	**All Paisley Target Population**	**All Paisley Target Population Screened**	**All Paisley Target Population Screened (%)**	**Ferguslie Park Area Target Population**	**Ferguslie Park Area Target Population Screened**	**Ferguslie Park Area Target Population Screened (%)**	**Ferguslie Park Area Target Population**	**Ferguslie Park Area Target Population Screened**	**Ferguslie Park Area Target Population Screened (%)**

**Male**	5,731	805	14.0	343	30	8.7	313	52	16.6

**Female**	5,567	1,043	18.7	287	20	7.0	267	96	36.0

**SIMD 1**	2,080	463	22.3	0	0	0.0	0	0	0.0

**SIMD 2**	1,845	363	19.7	0	0	0.0	0	0	0.0

**SIMD 3**	1,317	240	18.2	0	0	0.0	0	0	0.0

**SIMD 4**	2,146	334	15.6	0	0	0.0	0	0	0.0

**SIMD 5**	3,885	448	11.5	630	50	7.9	580	148	25.5

	**11,273**	**1,848**	**16.4**	**630**	**50**	**7.9**	**580**	**148**	**25.5**

The social marketing campaign generated 1,848 acceptances (16.4% of the target population), the acceptance rate in SIMD quintile 1, the least deprived SIMD quintile (22.3%, n = 463) was almost double that of SIMD quintile 5, the most deprived SIMD quintile (11.5%, n = 448). The Ferguslie Park area had the lowest uptake following the social marketing campaign (7.9% of the area's eligible population, n = 50).

The community development project produced an additional 148 acceptances, a quarter of all eligible individuals living in the Ferguslie Park area (eligible population = 580, all of which are within the most deprived SIMD quintile). Of the 148 acceptances, 52 (35.1%) attended follow-up screening at six months and were invited to take part in the qualitative study. Individuals expressing an interest in the qualitative study received written materials explaining the purpose of the study and, of these, 13 (26%) participated in the focus group discussions (6 (46%) male; 7 female).

### Data collection

Focus groups are an established method for accessing personal experiences and for facilitating more in-depth understandings of participants' views [16]. In particular, it has been suggested that focus groups are effective in encouraging participation from disempowered, excluded patient populations [17]. Although they may take many forms, the method essentially entails engaging a small group of participants in a group discussion, focused around a particular set of set of issues [16,18].

Two focus group discussions (n = 6, n = 7) were carried out, in order to explore the views and experiences of individuals recruited to the HaHP primary prevention study via the community development project. The focus group schedule asked participants to reflect on the engagement methods used in both the social marketing campaign and the community development project (for example, questions included "in what way were you approached to come along to the Ferguslie Screening?" and "what was it that made you decide to come along for a health check?"). Group discussions were carried out in a private room in a community centre in Ferguslie Park. Anonymity and confidentiality were assured and participants were encouraged to be frank and honest with their contributions. The meetings lasted approximately one hour and were audio-recorded, with permission, using a digital machine. Both focus groups were facilitated by a researcher with experience in community development and health improvement.

### Data Analysis

Focus groups were audio-recorded, transcribed verbatim by a member of the research team and analysed using thematic analysis (one of the most common approaches to analysing qualitative data, especially within the field of health-related research) [19]. Thematic analysis involves coding respondents' talk into categories that summarise and systemise the content of the data [20]. In this instance categories were derived from the data (rather than the prior theoretical framework of the analysts). The advantage of this approach in this context is that the analysis provides a useful summary of participants' views and experiences and an overview of the range and diversity of the ideas presented. The quality of the analysis was ensured through the close collaboration of multiple analysts (CH, RS, MG) with varied backgrounds throughout the process [20-22].

The three analysts read through each transcript several times, in order to familiarise themselves with the data and identify key issues and initial codes. Initial codes were identified by each of the analysts independently and data relevant to each code was collated. In a subsequent team meeting this coding was discussed and refined. Multiple coding, such as that adopted here, has been advocated as a way in which to refine coding frames and enhance rigor within qualitative studies [23]. The coded data were then sorted into potential themes, again by the three analysts independently, using a process whereby the identified themes were compared across the data. Interpretations of identified themes were discussed within the team, and re-assessed and re-interpreted as necessary. Direct quotes from the data were grouped under thematic headings [24] providing a clear illustration of each theme and also some indication of the frequency with which each theme was addressed. Finally, the themes were refined through investigation both of similar and anomalous examples [25]. Towards the end of the study no new themes emerged, which suggests that the major themes had been identified. A qualitative data indexing package (Atlas.ti) was used to facilitate coding and retrieval of the data. Quotations were chosen to illustrate particular points and are identified in the text by an anonymised code (indicating respondent number and the focus group discussion they participated in).

The study was approved by the South Greater Glasgow NHS Research Ethics Committee.

## Results

The focus group discussions highlighted a number of reasons for the lower uptake of risk screening in response to the social marketing campaign amongst individuals living in areas of high SED and a number of ways in which the community development project overcame these barriers. These have been categorised into four main themes: (1) processes of engagement; (2) issues of understanding; (3) design of the screening service and (4) the priority accorded to screening. In the results, presented below, the authors have edited some of the respondents' West of Scotland dialect for presentational purposes and ease of understanding.

### Processes of engagement

The most immediate barrier to recruitment for many residents of Ferguslie Park was that the initial letter inviting them for screening did not reach them, as a result of inaccurate address information. The majority of focus group participants acknowledged this difficulty and spoke frankly about why individuals living in Ferguslie Park might be difficult to locate; citing escaping debt and committing benefit fraud as reasons:

*Folk like that are not wanting to be on any list, anywhere, they think they're all after them for whatever it is - debt, giros, so they're not going to go for anything like this. They probably think you're wanting to get them off the (state) benefits (laughter of group) *(Respondent 5, Group 1)

There was also consensus that mass mailings are ineffective in Ferguslie Park; being perceived as 'junk mail' to be immediately discarded:

*If you get a letter in you just go 'oh more junk mail' and you throw it in the bin *(Respondent 5, Group 1)

In contrast, interviewees suggested that social networks, extending beyond that of the immediate family, were highly effective methods for communication and for dispelling mistrust and misunderstanding of new services in the area:

*Aye you just have to bang the jungle drums to get the word out there- best way! (laughter) *(Respondent 2, Group 1)

Both focus groups were very positive about the processes of engagement employed by the community development project, which included on-street interviewing, door-to-door calling, peer referral and involvement of the outreach workers in local events. Participants repeatedly suggested that it was the face-to-face nature of the community development project that was pivotal to their engagement and recruitment:

*I think we all came because of the approach, yeah *(Respondent 4, Group 1)

In particular, participants felt that the enthusiasm of the outreach workers and their ability to establish rapport and engender trust with the target population was essential to recruitment:

*Meeting the woman *(community outreach worker) *she was great, I wouldn't have bothered otherwise *(Respondent 3, Group 2).

*They made you feel comfortable the lassies *(community outreach workers) (Respondent 4, Group 1)

*What made me come along to the screening was that I got to know the workers *(Respondent 3, Group 1)

### Issues of understanding

Focus group participants expressed a lack of knowledge and understanding about both CHD and their individual risk of developing it. Several participants stated that they did not engage with the initial (mailed) invitation to screening because they felt they were in good health and did not require risk screening:

*I just didn't feel I needed it*, (screening) *I just didn't feel... ill *(Respondent 4, Group 2)

The information contained within the social marketing mass mailing failed to overcome these general issues of knowledge and understanding regarding CHD. In addition, participants felt there was of a lack of clarity in the information provided in terms of the process and value of screening. In particular, participants suggested that they were left unclear as to what participation would involve and what the benefits of screening would be for those who accepted the invitation:

*I didn't know what it was about, I didn't know if they'd have me on a treadmill or anything like that and I wasn't wanting that *(Respondent 1, Group 1)

In contrast, the direct face-to-face contact employed by the community development project meant that the community outreach workers were able to explain to prospective participants what was involved and what the participant could expect from the screening service, as well as the potential benefits to the participant. This was recognised in both groups as being vital to recruitment:

*The fact that you were getting everything explained to you so it didn't matter that you couldn't read the glossy leaflet *(social marketing material) (Respondent 3, Group 1)

### Design of the screening service

In order to accept and take-up the invitation for screening, participants were required to make an appointment over the telephone and then attend the screening location at this time. Focus group participants felt that this procedure was demanding, inflexible to individual needs and placed too much responsibility on the individual:

*It was too much hassle, trying to arrange all that and go up to town *(screening location), *to take time off your work and that *(Respondent 3, Group 1)

*I couldn't make the appointment and didn't know if I could get another *(Respondent 5, Group 2)

These issues were considered to be particularly acute for individuals, such as residents of Ferguslie Park, with a range of competing issues (for example housing, violence, drug abuse and addiction) that may be of higher priority than organising a screening appointment:

*There's so many issues in Ferguslie, in people's lives, whether it's the drugs, the alcohol, violence *(Respondent 1, Group 1)

*I got the stuff through and I actually called up and got an appointment through but it wasn't a good time for me, I couldn't commit to the appointment time, not with what I've got on my plate *(Respondent 5, Group 1)

In contrast, the community development project ran a series of drop-in sessions where appointments were not required. Focus group participants indicated that this flexible approach was more suitable to the competing priorities of the local residents and had a major impact on recruitment, retention and quality of care:

*Ye need a variety of times, you're maybe watching the grand weans *(grandchildren) *because something mental has happened the night before *(Respondent 5, Group 2)

In addition, both focus groups identified the importance of the longer appointment times available through the community development project which allowed participants the time to receive, understand and ask questions about the results of their risk screening:

*It wasn't like the doctors they chase you out the door. Because you were there for as long as you wanted (*Respondent 2, Group 1)

Participants in both focus groups also discussed their prior negative experiences with both primary and secondary care services, describing how traditional healthcare settings made them feel uncomfortable. These experiences were directly cited as reasons to be sceptical of the screening service:

*You're uncomfortable, it's the way they *(doctors and nurses) *fire questions at you *(Respondent 2, Group 1)

*You're on eggshells the whole time *(when with doctor) *aren't you? *(Respondent 5, Group 1)

The community development project used an informal (non-health service) location for screening, overcoming the issues that some participants may have with traditional healthcare settings and staff. In particular the staff were repeatedly described as non-judgmental and non-patronising:

*The fact that it's not formal and you were never getting lectured to *(Respondent 3, Group 1)

*You could actually speak to them *(community outreach workers and project nurses). *When I go up the hospital- the nurses and that talk to me, I'll say anything to them *(Respondent 2, Group 1)

*I think you never felt as though you were being patronised *(Respondent 3, group 1)

## Discussion

### Summary of main findings

This qualitative study has provided valuable insight into the barriers and facilitators to engaging deprived populations in a CHD primary prevention programme. Participants suggested that they had a lack of knowledge about and awareness of CHD; few considered themselves to be in an at-risk group. Consequently many prioritised other social and environmental issues ahead of CHD primary prevention. Moreover, many respondents had previously had a negative experience of engaging with health services. The mass mailing approach adopted in the social marketing campaign not only failed to address the former issues, but may have exacerbated the latter; most respondents in our study reported a distrust of postal correspondence.

The recruitment success of the community development project illustrated that these issues were not insurmountable. The focus group respondents reported that the face-to-face recruitment had played a crucial role in changing their attitudes toward the intervention and altering their behaviour. Our finding, that individuals are more likely to engage when approached to do so in person outside of a health care setting, would support the use of multiple recruitment strategies operating in parallel in order to ensure adequate reach and equitable recruitment.

### Relevance of findings

Our study directly addresses the challenge faced by policy-makers trying to implement effective primary prevention interventions without widening health inequalities, by examining and presenting the experiences of participants, from an area of high socioeconomic deprivation, regarding engagement with a primary prevention programme.

There is a paucity of literature exploring the barriers to engaging socio-economically disadvantaged populations in CHD primary prevention interventions against which to compare our findings. Qualitative studies in other disease areas have called into question the use of mass mailings as an equitable approach to recruiting patients to medical trials or interventions [26-28], particularly when recruiting participants from socio-economically disadvantaged populations [29]. There is evidence that individuals from socio-economically disadvantaged populations require additional approaches to recruitment and that, as demonstrated by the community development project, uptake can be increased through recruitment within the community setting [30]. Similarly, a number of studies exploring the uptake of cancer screening services have noted, as we have found, that social and environmental barriers to engaging socio-economically deprived populations can be overcome by increasing the accessibility and flexibility of service design [31-33].

### Strengths and limitations

Qualitative research methodologies are increasingly recognised as being valuable in furthering understanding of patients' cardiovascular health related behaviours [34]. Within this study, the use of focus groups allowed us to explore in detail participants' perceptions of the social marketing and community development approaches to recruitment, in order to understand why SED groups did not engage with the intervention following the social marketing campaign. The sample size, which was small compared to that for quantitative studies (13 participants took part in two focus groups), was sufficient to achieve saturation with similar issues arising in both focus group discussions. As such, the key lessons from the study should be generalisable to other SED populations. These relate to the need to identify emergent inequalities when primary prevention interventions are being delivered, to explore perceived barriers and facilitators to engaging hard to reach populations and to tailor recruitment strategies to overcome these barriers.

## Conclusions

Primary prevention is being actively pursued as a public health policy to reduce the growing burden of chronic disease in the UK. However primary prevention interventions that adopt population-wide recruitment strategies may exacerbate existing health inequalities by failing to recruit socio-economically disadvantaged groups. The community development project has shown that these barriers to engaging socio-economically disadvantaged groups can be overcome by adopting face-to-face recruitment within the community setting to deliver a flexible, reactive service. This type of personal approach allows for exploration of participant's health beliefs and attitudes and facilitates the delivery of tailored information, thus engendering trust. This study suggests that individuals from across all socio-economic strata can be engaged and recruited into primary prevention. This is necessary to ensure that primary prevention programmes do not increase health inequalities.

## Competing interests

The authors declare that they have no competing interests.

## Authors' contributions

IF, HS and CH conceived of the study. HS conducted and recorded the focus groups. CH, RS and MG performed the thematic analysis of the transcribed focus groups. CH drafted the manuscript with significant input from MG and RS. PD performed quantitative analysis of recruitment yields. KM, AS, CM, FM, HC, PD, PM and IF participated in the study design, coordination and helped draft the manuscript. All authors read and approved the final manuscript.

## Pre-publication history

The pre-publication history for this paper can be accessed here:

http://www.biomedcentral.com/1471-2458/10/391/prepub
